# Functional Reconstitution of Membrane Proteins Derived From Eukaryotic Cell-Free Systems

**DOI:** 10.3389/fphar.2019.00917

**Published:** 2019-08-30

**Authors:** Srujan Kumar Dondapati, Henning Lübberding, Anne Zemella, Lena Thoring, Doreen A. Wüstenhagen, Stefan Kubick

**Affiliations:** ^1^Fraunhofer Institute for Cell Therapy and Immunology (IZI), Branch Bioanalytics and Bioprocesses (IZI-BB), Am Mühlenberg, Potsdam, Germany; ^2^Faculty of Health Sciences, Joint Faculty of the Brandenburg University of Technology Cottbus – Senftenberg, The Brandenburg Medical School Theodor Fontane, University of Potsdam, Potsdam, Germany

**Keywords:** membrane proteins, Sf21 lysates, microsomes, cell-free protein synthesis, proteoliposomes, transporter, ion channel, pump

## Abstract

Cell-free protein synthesis (CFPS) based on eukaryotic *Sf*21 lysate is gaining interest among researchers due to its ability to handle the synthesis of complex human membrane proteins (MPs). Additionally *Sf*21 cell-free systems contain endogenous microsomal vesicles originally derived from the endoplasmic reticulum (ER). After CFPS, MPs will be translocated into the microsomal vesicles membranes present in the lysates. Thus microsomal membranes offer a natural environment for *de novo* synthesized MPs. Despite the advantage of synthesizing complex MPs with post translational modifications directly into the microsomal membranes without any additional solubilization supplements, batch based *Sf*21 cell-free synthesis suffers from low yields. The bottleneck for MPs in particular after the synthesis and incorporation into the microsomal membranes is to analyze their functionality. Apart from low yields of the synthesized MPs with batch based cell-free synthesis, the challenges arise in the form of cytoskeleton elements and peripheral endogenous proteins surrounding the microsomes which may impede the functional analysis of the synthesized proteins. So careful sample processing after the synthesis is particularly important for developing the appropriate functional assays. Here we demonstrate how MPs (native and batch synthesized) from ER derived microsomes can be processed for functional analysis by electrophysiology and radioactive uptake assay methods. Treatment of the microsomal membranes either with a sucrose washing step in the case of human serotonin transporter (hSERT) and sarco/endoplasmic reticulum Ca2+/ATPase (SERCA) pump or with mild detergents followed by the preparation of proteoliposomes in the case of the human voltage dependent anionic channel (hVDAC1) helps to analyze the functional properties of MPs.

## Introduction

Cell-free synthesis of proteins (CFPS) offers several advantages over traditional approaches. In CFPS, the protein of interest is synthesized by using the translation machinery, retained in the material formed by the lysis of cells (Carlson et al., 2012; Zemella et al., 2015). Cell-free protein synthesis (CFPS) is a fast and user-friendly technique, where the protein production process is completed within 2h and proteins can be analyzed immediately after the synthesis. Additionally CFPS is an open and flexible system and allows the facile modification of components related to protein translation. Thus, for efficient CFPS, supplementation of reaction components such as amino acids, RNA polymerase, salts, and energy components can be actively monitored and manipulated specific to each individual protein. Additives in CFPS substantially influence both the folding and quantity of functional protein.

For human MPs, a cell-free system based on eukaryotic lysates is ideal as this system will ensure properly folded and post-translationally modified proteins. Additionally, the MPs get translocated though the translocon machinery and are thereby incorporated into the ER derived microsomes present in the eukaryotic lysates. Thus, one can use the protein charged microsomes for downstream functional analysis (Carlson et al., 2012). In this context CFPS promotes the engineering of proteins and increases the functionality of MPs. Furthermore, CFPS enables the production of difficult-to-express proteins and toxic proteins (Orth et al., 2011; Bechlars et al., 2013; Thoring et al., 2017) as well as the synthesis of modified proteins containing non-canonical amino acids (Quast et al., 2015). Although several MPs were expressed using insect based cell-free systems, very few reports were shown demonstrating the functionality of the expressed proteins. Therefore developing a more efficient synthesis method combined with a more flexible functional screening assay will help to study in detail the protein properties and screen pharmaceutical targets.

Through this communication, we attempt to show how the proteins incorporated in ER derived vesicles after synthesis by eukaryotic cell-free lysates can be processed for functional analysis by different methods. For this, we selected three different MPs with diverse structures and functions (Bayrhuber et al., 2008; Coleman et al., 2016). First, human voltage dependent anionic channel 1 (hVDAC1) was synthesized using a *Sf*21 based cell-free system. hVDAC1 is the most abundant protein in the outer membrane of mitochondria and plays an important role in cancer due to its close connection with mitochondria based apoptosis (Coleman et al., 2016). Second, the human serotonin transporter (hSERT), a sodium symporter with 12 transmembrane domains was synthesized in the same based cell-free system. This protein plays a major role in neurotransmission (Coleman et al., 2016). Third, sarco/endoplasmic reticulum Ca2+/ATPase (SERCA) a type P ATPase membrane transport pump involved in cell Ca^2+^ signaling and homeostasis was analyzed from the native ER vesicles derived from the *Sf*21 lysate. SERCA utilizes the energy generated by ATP hydrolysis to pump two Ca^2+^ ions from the cytoplasm to the ER lumen of the cells (Tadini-Buoninsegni et al., 2006; Sacconi et al., 2013; Tadini-Buoninsegni et al., 2015). SERCA pump is often used as a drug target, particularly in the treatment of cancer due to its role in Ca^2+^ regulation.

For ion channel analysis, the planar bilayer electrophysiology (PLB) technique was used for functional analysis. This approach is widely reported for the analysis of a wide range of ion channels (Banerjee and Nimigean, 2011; Carnarius et al., 2012; Zakharian, 2013; Dondapati et al., 2014; Jo et al., 2017). Among MPs, transporters mediate the translocation of a variety of substrates across biological membranes. In the case of transporters, radiolabeled assays are widely used as an important technique for functional analysis (Gros and Schuldiner, 2009; Coleman et al., 2016). In this assay format, lipid vesicles harboring the transporter protein are incubated with the radiolabeled substrate solubilized in the buffer containing the ion of choice. After incubation, the vesicles are washed and analyzed for the radioactivity. In contrast to ion channels, pumps have a drawback of low turnover rate and are difficult to analyze by traditional electrophysiology techniques. An alternative and relatively new approach for these type of proteins is solid supported membrane (SSM) based electrophysiology (Bazzone et al., 2013; Wacker et al., 2014; Barthmes et al., 2016). This technique uses gold sensors with a very large area of mm size which allows immobilizing several proteins. As a result a cumulative current from all the transporters (10^9^) in real time due to charge translocation *via* capacitive coupling through supported membrane electrode can be measured simultaneously with a high signal to noise ratio.

The overall aim of this research communication is to show how MPs obtained from eukaryotic *Sf*21 cell-free derived lysates can be processed for functional analysis by different methods.

## Materials and Methods

### Coupled Batch Based Cell-Free Synthesis of hSERT and hVDAC1

*Sf*21 lysates preparation and coupled CFPS in general have been described previously and explained in **Sup 1.1** (Brödel et al., 2013; Quast et al., 2015). Information about the DNA template was described in **Sup 1.2**. Coupled transcription–translation reactions were performed in a batch mode format. Protein synthesis was mainly performed at optimal reaction temperature in a thermomixer (Thermomixer comfort, Eppendorf, Hamburg, Germany) with gentle shaking at 500 rpm for 2h. Reaction volumes of 50µl were composed of 40% (v/v) *Sf*21 cell lysate, 100 mM of each canonical amino acids, nucleoside triphosphates (1.75 mM ATP, 0.30 mM CTP, 0.30 mM GTP, and 0.30 mM UTP), 60 nM vector DNA, and 1 U/ml T7 RNA-polymerase (Agilent, Waldbronn, Germany). To monitor protein quality and quantity, reaction mixtures were supplemented with 14C-labeled leucine (specific radioactivity 75.0 dpm/pmol). No template controls (NTC) were prepared in the same way as the samples with the exception of the DNA template which was replaced by RNase-free water. Standard conditions were used for optimal cap-dependent transcription–translation reactions (in the case of hSERT) in the coupled cell-free system based on extracts from *Sf*21 cells (Brödel et al., 2013). Repetitive translation reactions were performed for the enrichment of MPs into microsomal structures. After each translation step, translation mixture (SU) was separated into microsomal fraction (MF) and supernatant (SN) by centrifugation (16,000xg, 15 min, 4°C). Microsomal fraction was newly resuspended using freshly prepared translation mixture without vesicles to start a new translation cycle and thereby increasing the concentration of protein per microsomes (Quast et al., 2016; Thoring et al., 2016).

### Quantitative Determination of Protein Yield and Qualitative Analysis

For yield determination of radiolabeled proteins hot TCA precipitation was used. First, samples were fractionated into reaction mix, supernatant and microsomal fraction. Supernatant and microsomal fraction was obtained by centrifugation of the reaction mix at 16,000xg for 15 min at 4°C. five microliters of each fraction was transferred into a glass tube and 3 ml of 10% TCA/2% Caseine hydrolysate was added. The mix was boiled for 15 min in an 80°C water bath and afterwards chilled on ice for 30 min. Precipitated radiolabeled proteins were captured on a silica membrane filter (Macherey Nagel) using a vacuum filtration device (Hoefer). Dried membrane filters were placed into a scintillation tubes and 3 ml of scintillation cocktail was added and tubes were incubated for 1 h under modest agitation. For measurement of the radioactivity a LS6500 Multi-Purpose scintillation counter (Beckmann Coulter) was used.

Cell-free synthesized proteins were further analyzed by SDS-PAGE and autoradiography. Therefore, 5 µl of reaction mix, supernatant, and microsomal fraction was precipitated with 45 µl MilliQ water and 150 µl ice cold acetone, respectively. Samples were incubated for at least 15 min on ice and afterwards samples were centrifuged at 16,000xg for 10 min and 4°C. Acetone was removed completely and pellet was dried at 45°C for 1 h in a thermomixer. Dried pellets were resuspended in 1x LDS (NuPAGE LDS sample buffer supplemented with 50 mM DTT). Afterwards samples were separated on a 10 % Bis-Tris pre-cast NuPAGE SDS gel for 75 min at 150 V. SDS gels were stained using Coomassie blue solution (SimplyBlue SafeStain, Life Technologies) according to the manufacturer’s instructions. Later, SDS-gels were transferred on a Whatman paper and dried for 60 min at 70°C using a Unigeldryer 3545D, Uniequip). Autoradiography of radiolabeled proteins was performed by incubation of dried SDS-gels on a storage phosphor screen (GE Healthcare) for several days. Analysis of the screen was performed using a Typhoon TRIO+ Imager (GE Healthcare).

### Preparation of Proteoliposomes

After cell-free synthesis of hVDAC1, 20 µl of microsomes harboring the hVDAC1 was mixed with 20 µl DOTAP (10mM dissolved in Triton X-100) at room temparature for almost 15min. Later Biobeads-SM2 were added and incubated at room temperature for 30 min (Lévy et al., 1990; Thoring et al., 2017). Finally the supernatant was collected from the mixture without touching the biobeads.

### Planar Bilayer Electrophysiology

**Figure 1:**
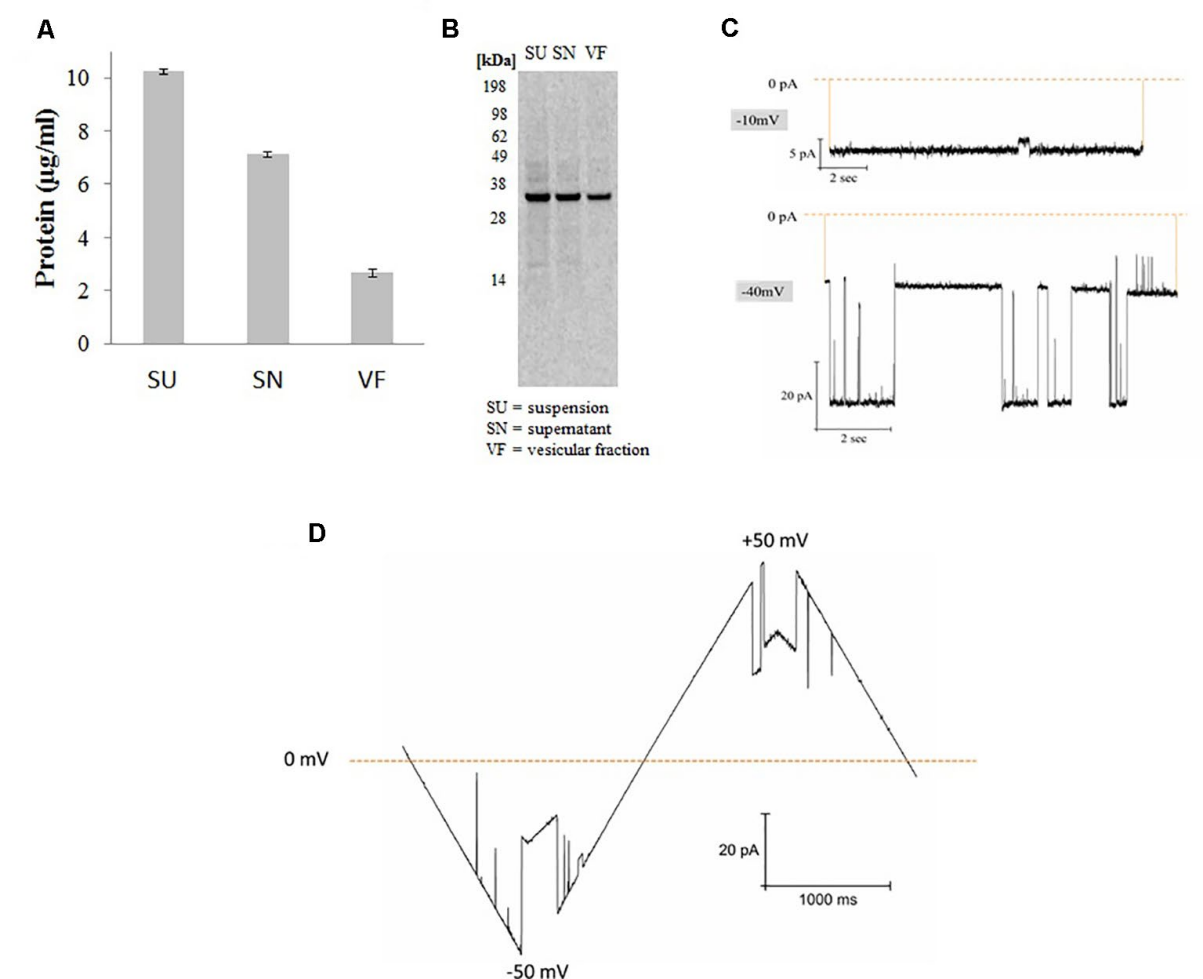
Synthesis and functional analysis of cell-free synthesized hVDAC1. **(A)** Quantification was performed by hot TCA precipitation of 14C Leucine labeled protein. **(B)** The cell-free reaction was separated using a 10% SDS polyacrylamide gel. **(C)** Functional analysis of proteoliposomes derived from microsomes harboring hVDAC1 at -10 and -40 mV. **(D)** Current response to a voltage ramp applied to a single hVDAC1; the membrane voltage was changed linearly between −50 and +50 mV.

Planar bilayer experiments were performed as explained previously (Dondapati et al., 2014; del Rio Martinez et al., 2015; Baaken et al., 2011) **Figures 1A, B**. Lipid bilayers were formed from 1,2‐diphytanoyl‐sn‐glycero‐3‐phosphocholine (DPhPC) (Avanti Polar Lipids, Albaster, AL, USA). Lipids were dissolved in octane (Sigma Aldrich, Munich, Germany) at a concentration of 10 mg/ml. 10mM HEPES, 150mM CaCl2 (Sigma Aldrich (Fluka), Munich, Germany) pH 7.45 was used as an electrolyte. Five microliters of the proteoliposomes mixed with 10µl of 1M sorbitol was added to the chamber containing the buffer and waited for the response. For blockage studies, poly (ethylene glycol) PEG‐1000, Mw = 950-1050 g/mol (FLUKA, Sigma Aldrich, Munich, Germany) were dissolved in the electrolyte solution to a concentration of 1 mg/ml and 10 μl of this solution was added to the 180 μl electrolyte in the measurement chamber for recordings. For current measurements, voltage ramp protocol as well as individual voltage steps were applied to analyze the functional properties.

### SSM-Based Electrophysiology

All the electrogenic current measurements were performed by the SURFE²R N1 (Nanion Technologies GmbH) at room temperature. All the salts were purchased from Sigma Aldrich. ATP was purchased from Life technologies. For thiolation of the chips, 1-octadecanethiol was purchased from Fluka. Electrogenic current transient measurements were performed by using solid-supported lipid membrane (SSM) technique. For forming SSM,1-Octadecanethiol was purchased from Fluka for forming monolayer over the gold surface by using Au-thiol dative bonding. Later lipid monolayer is formed from the diphytanoyl phosphatidylcholine (DPhPC) purchased from Avanti Polar lipids, USA. Next the chip surface was incubated with the sample by centrifugation at 3,000xg for 30min. Concentration jump measurements were performed by quick jump from deactivation buffer to activation buffer followed by deactivation buffer (Bazzone et al., 2013; Wacker et al., 2014; Barthmes et al., 2016). Transient currents due to charge transfer were measured by rapid exchange of deactivation and action buffers.

### Radioactive Uptake Assay

After the repetitive batch based synthesis reaction (3 X 50 µl) as explained in the materials, centrifugation was done at 16,000xg for 10 min at 4°C. The pellet containing the microsomal fraction was washed by resuspending in 50 µl of uptake buffer (10mM HEPES, 200mM NaCl, 320nM [14C] 5-HT, pH 7.5 adjusted with Tris) and the sample was split into 2 X 23 µl and centrifuged again for 10 min at 16,000xg at 4°C. Then the pellet was resuspended with 23 µl of uptake buffer containing 320 nM [14C] 5-HT (Perkin Elmer, USA) and incubated for optimal time period (0, 5, 10, and 15min) at 37°C (doublets for each point in time). Later 35 µl of the ice cold stop buffer (5 mM HEPES, 210 mM KCl, pH 7.5 with Tris) was added to the mixture and centrifugation was done at 16,000xg for 10min at 4°C. Later the pellet was resuspended in 23µl of the stop buffer and centrifugation was done one more time for 16,000xg for 10min at 4°C. Later the pellets containing the washed microsomes were resuspended in 23 µl of the PBS with 0.2% (w/v) Brij-35. Incubation was done for 15min with subsequent sonication. Finally scintillation count of 20 µl of the samples was measured (after addition of 3 ml scintillation liquid to sample in scintillation tube and incubation at room temperature for 1 h). All the control measurements were performed similarly with microsomes after cell-free synthesis without any plasmid (NTC).

## Results

### Synthesis and Functional Analysis of hVDAC1

hVDAC1 was synthesized in *Sf*21 based cell-free systems using a batch based 2h reaction. After synthesis, proteins were quantified by hot TCA precipitation as explained in the experimental section. For fractionation, total synthesis reaction which we mentioned as suspension (SU) is centrifuged at 16,000xg for 10 min and the pellet which we mentioned as vesicular fraction (VF) contains the protein incorporated microsomes is separated from the supernatant fraction (SN) which contains the cell-free components and other non incorporated protein as well as smaller microsomes. As shown in **Figure 1**, the protein yields were around 3 µg/ml in the pellet fraction containing the microsomes harboring the protein which is 30% of the total protein synthesized in the suspension. Analysis by SDS-PAGE electrophoresis followed by autoradiography shows a single clear full length band around 33 kDa corresponding to the molecular weight of hVDAC1 without any signs of truncated products. Based on our experience, we continued working with the microsomal fraction and prepared proteoliposomes as explained in the experimental section (Thoring et al., 2017).

**Figure 1** shows the functional analysis of the synthesized protein by planar lipid bilayer electrophysiology. The functionality of synthesized hVDAC1 was studied after reconstitution of the protein into a planar lipid bilayer. At -10 mV, the pore is completely open without any sub conductance states and at −40 mV, the channel shows currents switching between open and closed states with intermediate sub conductance states (**Figure 1C**). As shown in the **Figure 1D**, the channel, when inserted into the lipid bilayer, shows a linear slope in the current behavior at low voltages (< +/- 30 mV) corresponding to the opening of the channel and thereafter shows a shallow slope when the voltage is increased upto +/-50 mV corresponding to the closing of the channel. At higher voltages, the channel started to close completely and also shows sometimes step-like transitions between the closed and open states. This type of canonical voltage gating is a typical characteristic of VDAC1 when inserted into a planar lipid bilayer (Zimmerberg and Parsegian, 1986; Pavlov et al., 2005; Magrì et al., 2018). The currents were quite large and the current-voltage characteristics were the same in both polarities.

### Polymer Partitioning of hVDAC1

**Figure 2:**
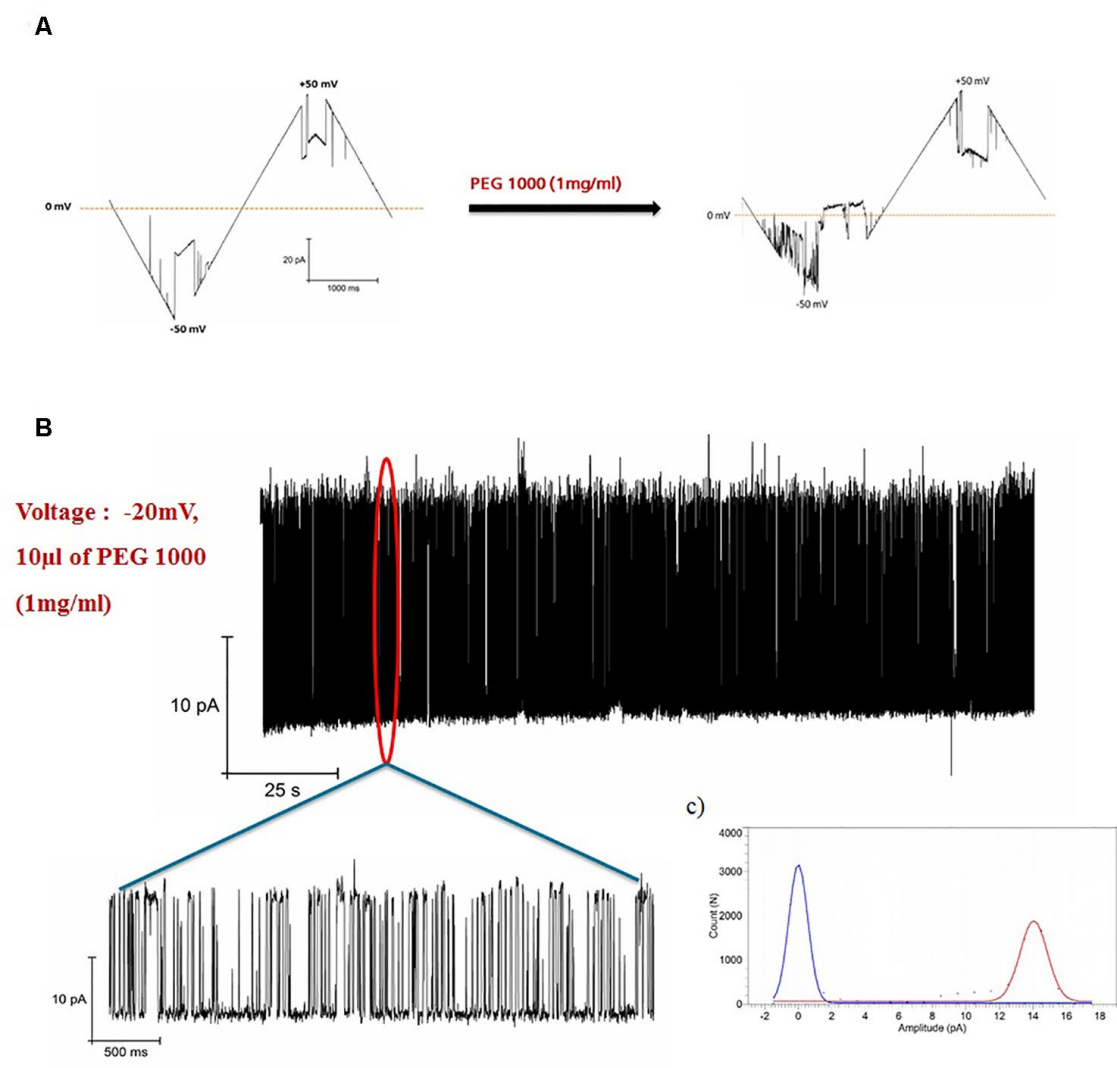
Blockage of hVDAC1 activity by PEG 1000. **(A)** Current response to a voltage ramp applied to a single hVDAC1 in the presence of PEG 1000. The membrane voltage was changed linearly between −50 and +50 mV. **(B)** hVDAC1 current blockades induced by the PEG1000 molecules at -20 mV. **(C)** Histogram of the current blockades showing the pore opening and closing events caused by the PEG1000 molecules. All measurements were performed in the presence of 10 mM HEPES, 150 mM CaCl2, pH 7.45.

The effect of PEG1000 on open pore of hVDAC1 is shown in **Figure 2**. When PEG1000 molecule is added to the pore, we saw rapid blocking events corresponding to the entry and exit of the PEG molecule across the pore. In all of our experiments, the channel was open at low voltages (+/-30 mV) and closed at higher voltages as shown in **Figure 1C**. Once the PEG molecule is added, there was a continuous flickering activity only in the negative voltages corresponding to the closure of the channel pore due to the interaction with the PEG molecule, whereas the channel was constantly open before the PEG addition. The blockade currents were reversible and markedly different from the gating induced by voltage alone at −40 mV (**Figure 1C**). Addition of PEG molecules increased the voltage sensitivity of hVDAC1 and as a result the channel starts to show the flickering activity starting at −10 mV and increasing until −50 mV (**Figure 2A**). When the flickering activity is measured at −20 mV in the presence of PEG molecules, we observed that there was a fast gating with transitions between two conductance states (**Figure 2B**). This type of voltage dependent behavior is absent in the case without the PEG molecule (**Figure 2A**). All point histograms of the gating behavior plotted at -20 mV showed strong and clear current peaks at 0mV and 14pA corresponding to two different conductance levels (**Figure 2C**). This behavior might be due to the rapid transition of PEG molecules along the channel pore.

### Cell-Free Synthesis and Functional Analysis of hSERT

**Figure 3:**
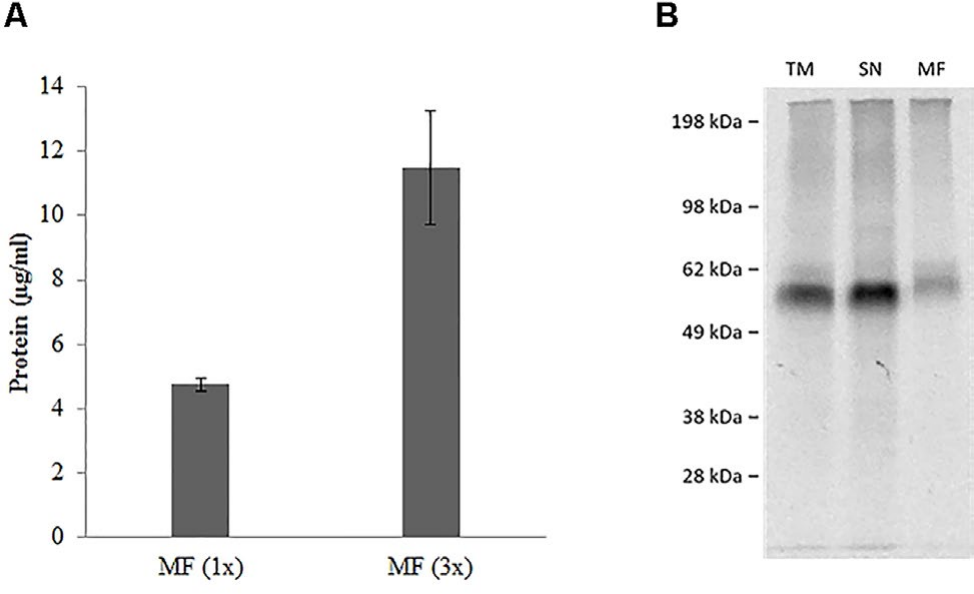
**(A)** Repetitive batch expression of NC-hSERT in *S*ƒ21 system. Protein yields in µg/mL determined by incorporation of [14C] leucine; samples were fractionated, the microsomal fraction (MF) was quantified; different bar colors represent different potassium concentrations in the TT mix; bars show mean protein yield of duplicate measurements with error bars showing the SD. **(B)** Autoradiography for batch based Expression of NC-hSERT in the *Sƒ*21 system. Samples were fractionated into translation mix (TM), supernatant 1 (SN) and microsomal fraction (MF).

hSERT was synthesized by using a coupled batch based *Sf*21 cell-free system. The yields were around 5 µg/ml within a 2 h reaction time and we used a batch based repetitive format for enriching the protein in the microsomal fraction (Thoring et al., 2016). Repetitive reactions were done for a total of three times (3 X 2 h). We can observe that the yields were around 11 µg/ml in the microsomal fraction (**Figure 3A**). We particularly concentrated on the vesicular fraction (MF) which contains the fully matured protein incorporated into microsomes. SDS-PAGE followed by autoradiography showed a clear band corresponding to the presence of protein (**Figure 3B**). The protein band in the case of the whole reaction mixture (TM) and the vesicular fraction (MF) has comparatively additional bands slightly above the molwt which might correspond to the glycosylation of the protein (Quast et al., 2015).

### Uptake of 5-Hydroxytryptamine Into Microsomes

The ability of the hSERT harboring microsomes to catalyze the transport of 14C-5-HT across the membrane depends on the sample pretreatment. Microsomes equipped with cell-free produced hSERT were washed with 0.25 and 2.5 M sucrose concentrations and compared with the 0 M sucrose treated samples (**Table 1**). Two different incubation times were tested with 14C-5-HT. Control samples without any SERT expression were treated in the similar manner. When we compare the radioactive scintillation counts, we noticed that there is not much significant difference between the NTC and 0 and 0.25 M sucrose treated microsomal samples. Only in the case of 2.5 M treated samples, we noticed there is significant increase in the counts up to 50% for 10 min and 25% for 20 min. The increase in the case of 0 and 0.25 M was around 5–10%.

**Table 1:** Radioactive Assay for hSERT from repetitive batch Synthesis with subsequent microsome washing.

	10 min		20 min	
Sucrose (M)	SERT	NTC	SERT	NTC
0	2634.54 +/- 47.55	2198.30 +/- 650.78	2383 +/- 95.78	2200.5 +/- 339.27
0.25	2354.40 +/- 219.156	2123.5 +/- 203.36	2455.09 +/- 157.78	2388.01 +/- 151.12
2.5	2402.5 +/- 209.24	1645.3 +/- 154.57	2019.4 +/- 115.28	1598.2 +/- 82.26

Next we tried to check the influence of centrifugation speed during fractionation of the synthesis reaction. In our observations with the protein yields we noticed that there is almost 10–50% of the protein retained in the SN fraction after fractionation by 16,000xg for 10 min. We further increased the centrifugation speed up to 50,000xg for 10 min and performed the radioactive uptake assay with 2.5 M sucrose concentration treatment. We noticed that when we increased the centrifugation speed during the fractionation, the increase in the radioactive counts was only 20% in comparison to 56% with lower centrifugation speed (**Sup Figure 1**).

Finally we chose 16,000xg centrifugation speed along with 2.5 M sucrose pretreatment are ideal conditions for our radioactive uptake assay measurement.

**Figure 4:**
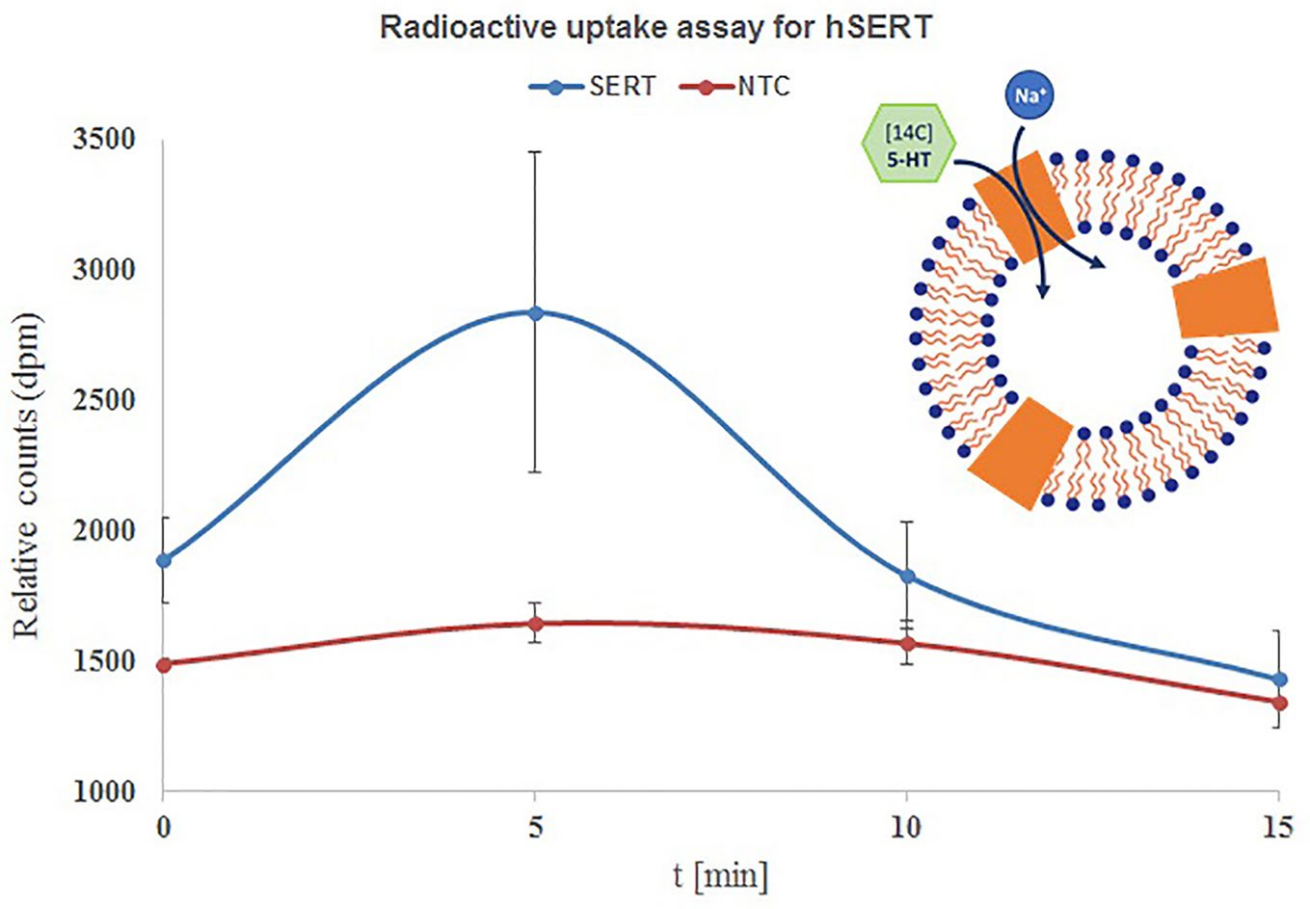
Time course uptake of 5-HT by hSERT synthesized by repetitive batch mode and incorporated in microsomes. Microsomal fraction from 3x repetitive batch synthesis of NC-hSERT was incubated with [14C] 5-HT in uptake buffer and stopped by addition of stop buffer at different points in time; centrifugation speed for fractionation was 16,000xg, sucrose concentration for microsome washing was 2.5 M; the detergent resuspended microsomal fraction of the second fractionation underwent liquid scintillation counting. Bars show mean counts of duplicate measurements with error bars showing the SD.

Next we have performed time dependent uptake measurements with the above applied conditions (Philippu and Matthaei, 1975; Ramamoorthy et al., 1992). The uptake of 14C-5-HT is quite fast and reached a maximum within 5 min and later started to decrease with 10 and 15 min. The highest accumulation was observed within 5 min and the uptake was increased from 26% at 0 min to 76% within 5 min and started to decrease to 16% and 6% within 10 and 15 min. The results are quite contrasting when we see the influence of incubation time, but overall observation is that the uptake is quite faster and completes within 5–10 min (**Figure 4**).

### Functional Analysis of SERCA

**Figure 5:**
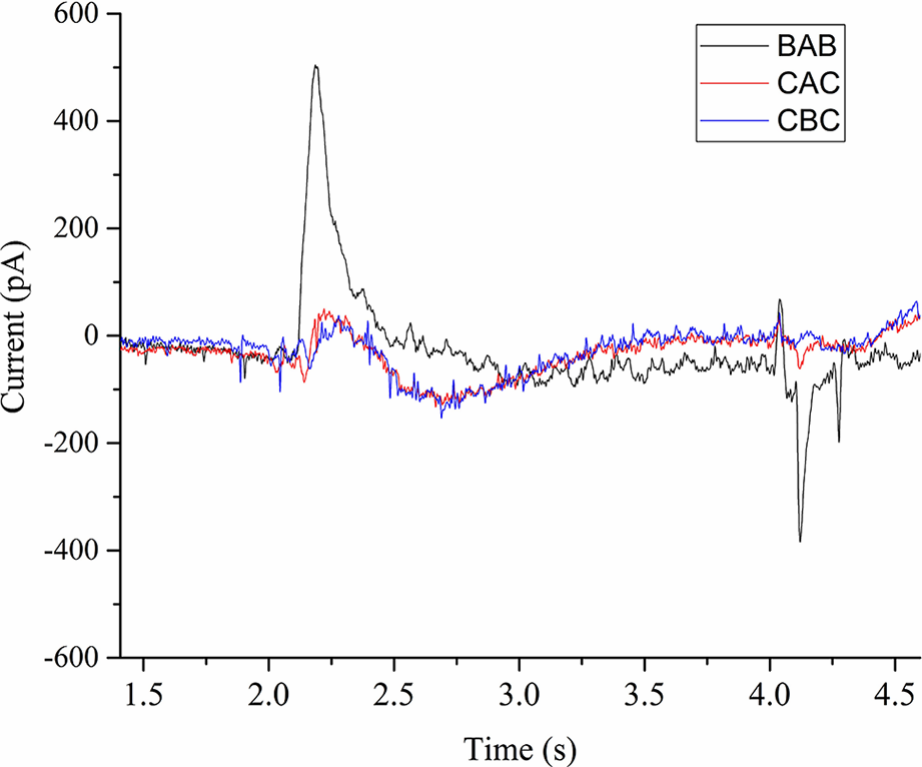
Current transients induced by 100µM ATP concentration jumps in the presence of (BAB and CAC). The non-activating solution (B) in the case of BAB contained 1 mM MgCl2, 0.3 mM CaCl2, 20 mM MOPS, 150 mM choline chloride, 0.2 mM DTT, and pH 7.0. The non-activating solution (C) in the case of CAC contained 1 mM MgCl2, 100 µM ATP, 20 mM MOPS, 150 mM choline chloride, 0.2 mM DTT, and pH 7.0. The activating solution (A) contained 1 mM MgCl2, 0.3 mM CaCl2, 100 µM ATP, 20 mM MOPS, 150 mM choline chloride, 0.2mM DTT, and pH 7.0. In the case of CBC, activating solution was buffer B and deactivation solution contains buffer C.

We performed current measurements for analyzing the SERCA activity on native ER derived vesicles from *Sf*21 cell-free systems. A 100 µM ATP concentration jump was carried out by including it only in the activation buffer (A) in the case of the BAB protocol. Due to rapid 100 µM ATP activation jump, the electrogenic event was recorded as a positive current peak around 500 pA followed by a deactivation peak (**Figure 5**). When the 300 µM Ca^2+^ concentration jump was carried out by including it in the activation buffer (A) in the case of the CAC protocol with C containing only the 100 µM ATP, There was an electrogenic event recorded as a current of around 70 pA. The amplitude resulting from the ATP jump was much higher compared to the Ca^2+^ jump.

We further performed experiments to check the influence of Ca2+ and ATP on the electrogenic signal. When we used the CBC protocol with B as activation buffer, the response was very minimal (60 pA). This confirms that either ATP or Ca2+ alone doesn’t generate an active signal. Next we checked the influence of ATP and Ca2+ on the activation peak by using buffer D as deactivation buffer (1 mM MgCl2, 20 mM MOPS, 150 mM choline chloride, 0.2 mM DTT, and pH 7.0). When we used the DCD protocol with C as activation buffer containing only ATP, we did not observe any signal. Next we used the DBD protocol to check the influence of Ca2+ alone and we did not notice any signal. Finally when using the DAD protocol, there was an active electrogenic signal of around 500pA. From these measurements, the electrogenic response shown in the **Figure 5** is a combined effect of ATP and Ca^2+^.

## Discussion

CFPS using batch based systems is the most commonly used eukaryotic cell-free reaction mode. On the one hand it is a very fast procedure (synthesis is usually completed within 2 h) and on the other hand proteins can be analyzed for their functionality within the same working day. As we noticed from the data presented in this report, the protein yields are comparatively low (Zemella et al., 2015). Repetitive batch based synthesis can be performed to increase the protein yield per microsome by using the same set of microsomes used in the first synthesis reaction for the next round of reactions (Quast et al., 2016; Thoring et al., 2016). Some of the advantages like fast synthesis, post translational modifications, incorporation into microsomes through the active eukaryotic translocon make them an ideal choice for synthesizing complex MPs harboring multiple transmembrane domains and displaying several post translational modifications (e.g., glycosylation). But proteins synthesized using batch based systems are limited in their functionality studies due to the difficulty in accessing the proteins incorporated in microsomes. Most of the functional assays are either limited to ligand binding studies or enzymatic activity measurements (Quast et al., 2016; Zemella et al., 2017). We tried to increase the scope of this method by offering different sample processing methods so that proteins present in the microsomes can be readily accessible and the functionality can be measured. For ion channels, planar bilayer technology is an ideal method for analyzing the functionality. This method is based on the reconstitution of a membrane protein onto a planar lipid bilayer followed by measuring the current activity from the reconstituted lipid bilayer. For a good reconstitution, the protein incorporated in the microsomes must be fused to the underlying planar lipid bilayer. We noticed that fusion of microsomes directly to the planar lipid bilayer is quite difficult and might be time consuming. In order to increase the fusion efficiency, we mixed artificial lipids dissolved in detergents to the microsomal membranes and prepared proteoliposomes by biobeads treatment. As a result, the fusion efficiency is increased and we can notice the protein activity specific to hVDAC1. PEG1000 was used for studying the partitioning of the hVDAC1 pore. Addition of PEG molecules disturbed the pore with a continuous flickering in the negative direction. This could be due to the presence of PEG molecules on the cis side and also negative charge of the PEG1000 at pH 7.45 (Pavlov et al., 2005; Tan et al., 2007; Gurnev et al., 2011). Functionality of hVDAC1 and polymer partitioning just shows that despite the protein synthesis in the batch based mode, sample processing plays a key role for long term measurements to study the single channel by electrophysiology.

The sucrose washing step played an important role for analyzing the functionality of the protein samples. We performed detailed zetasizer measurements of the washed microsomes assuming that the microsomes are present not as individual particles but more in agglomerations (Quast et al., 2016). We noticed when the microsomes are washed with PBS (without sucrose), there was a significantly large peak corresponding to the agglomerates. When we washed the microsomes after the synthesis with 0.25 M sucrose, the large peak was comparatively low and disappeared completely after washing with 2.5 M sucrose. We noticed that by treating with higher concentrations of sucrose, the agglomerates got separated into smaller particles or clusters which we noticed with a shift in the peak towards smaller diameters (**Sup Figure 2**). From these zetasizer observations combined with the radioactive accumulation measurements, we assume that with higher sucrose concentration, relatively more microsomal surface containing the protein is accessible to the substrate. To continue further and improve the assay standards, one may focus on increasing the protein per microsomes ratio. Further, if scaling up the reaction volume combined with an increase in protein yields, the difference in the radioactive accumulation between the NTC and the protein sample could be more significant. Additionally, optimizing the sodium concentrations in the uptake buffer might further increase the transport of the substrate into the microsomes. Optimizing the radioactive uptake conditions further, is an essential prerequisite to work with the blockers specific to hSERT for pharmacological profiling. Time dependent accumulation measurements showed a bell shape curve with the highest accumulation reaching within 5–10 min and thereafter started decreasing rapidly. This could be due to the significant efflux of the preaccumulated substrate and due to the large concentration gradient of the sodium ions developed inside the microsomes with time which exceeds the inward directed concentration gradient resulting in pushing back the substrate outside the microsomes (An et al., 2014). Working with different concentrations of 5-HT might change the time dependent profile of the radioactive assay.

For studying the transporter proteins by SSM-based electrophysiology, one need not reconstitute the proteins like in PLB electrophysiology. In this case, protein decorated microsomes can be directly added to the SSM lipid layer. We observed that the response is completely absent and it is very difficult to analyze the functionality. We applied the same sucrose treatment protocol used in the case of hSERT for removing the cytoskeletal components surrounding the microsomes so that the substrate or ions can access or pass the pump and generate the electrogenic signal. We are able to detect the Ca2+ dependent ATP response from the native microsomes corresponding to the SERCA pump. In future this technology may be applied to more electrogenic transporters expressed by cell-free synthesis for developing a pharmacological profile for each individual protein.

Finally we demonstrated how sample processing of the protein embedded in microsomes derived from the cell-free system plays an important role for studying the functionality of MPs. This article caters to the researchers who would like to utilize the full potential of eukaryotic cell-free systems for studying their protein’s functionality.

## Data Availability

The raw data supporting the conclusions of this manuscript will be made available by the authors, without undue reservation, to any qualified researcher.

## Author Contributions

SKD is the first author who planned the whole research and wrote the article. He did all the experiments related to hVDAC1 and SERCA. HL has done hSERT functionality measurements. AZ contributed to hSERT experimental design. LT was responsible for lysate preparation. DW contributed to lysate preparation and functionality studies design. SK was the overall supervisor.

## Funding

This work is supported by the European Regional Development Fund (EFRE) and the German Ministry of Education and Research (BMBF, No. 031B0078A).

## Conflict of Interest Statement

The authors declare that the research was conducted in the absence of any commercial or financial relationships that could be construed as a potential conflict of interest.
