# Supplementary material for: Functional Reconstitution of Membrane Proteins Derived From Eukaryotic Cell-Free Systems
Source: Front Pharmacol. 2019 Aug 30;10:917. doi: 10.3389/fphar.2019.00917 (PMC6728924; doi:10.3389/fphar.2019.00917)
Supplement: Supplementary file 1 [file DataSheet_1.docx]

**Supplementary Information**

**Functional reconstitution of membrane proteins derived from eukaryotic cell free lysates**

**Srujan Kumar Dondapati^1^, Henning Lübberding^1^, Anne Zemella^1^, Lena Thoring^1^, Doreen A Wüstenhagen ^1^, Stefan Kubick^1*^**

**^1^ Fraunhofer Institute for Cell Therapy and Immunology (IZI), Branch Bioanalytics and Bioprocesses (IZI-BB), Am Mühlenberg 13, D-14476, Potsdam, Germany**

**^*^** [**stefan.kubick@izi-bb.fraunhofer.de**](mailto:stefan.kubick@izi-bb.fraunhofer.de)

**1.1. Preparation of *Sf*21 lysates**

*Sf*21 lysates were prepared as explained in [22]. In brief, *Sf*21 cells were grown exponentially at 27°C in well-controlled fermenters using chemically defined, serum-free media (Insect-XPRESS medium, Lonza). Cells were collected in exponential growth phase at a density of approximately 4.0×10^6^ cells/mL by centrifugation at 200× g for 5 min. The resulting cell pellets were washed twice and resuspended with a buffer consisting of 40 mM HEPES-KOH (pH 7.5), 100 mM NaOAc and 4 mM DTT to a final cell density of approximately 2 × 10^8^ cells/mL. Cells were disrupted mechanically by passing the cell suspension through a 20-gauge needle using a syringe and the homogenate was centrifuged at 10,000× g for 10 min in order to remove the nuclei and cell debris. Supernatants were applied to a Sephadex G-25 column (GE Healthcare, Freiburg, Germany), equilibrated with the above mentioned resuspension buffer, and the elution fractions (each 1 mL) with an RNA content above an absorbance of 100 at 260 nm were pooled. Finally, cell lysates were immediately shock-frozen in liquid nitrogen and stored at −80°C to preserve maximum activity.

**1.2. Vectors used for the cell-free synthesis**

All plasmids used herein contain the T7 promoter (T7-P) and terminator sequences (T7-T) upstream and downstream of the ORFs, respectively. For hVDAC1, we used a construct without any 5´-UTR elements (CrPV-IRES) upstream of gene. For hSERT, we used a construct with an CrPV-IRES upstream of the gene. In the case of hSERT, initiator AUG-codon was replaced to a GCU-codon. All the DNA templates were inserted into pMX vector manufactured by GeneArt (ThermoFisher Scientific).

1) Vector constructs (N-hVDAC1)

**T7-T**

**hVDAC1**

**T7-P**

2) Vector construct (NC-hSERT)

**T7-T**

**hSERT**

**CrPV-IRES**

**T7-P**

**Figure 1**

**Sup Figure 1. Centrifugation speed optimization for radioactive assay with microsomal fraction from batch Expression of NC-hSERT in *Sf*21 System.** Fractionation of 3x repetitive batch synthesis of NC-hSERT was done at two different centrifugation speeds 16000 g and 50000 g; microsomal fraction was incubated with [14C] 5-HT in uptake buffer and stopped by addition of stop buffer after 10 min; in detergent resuspended microsomal fraction of second fractionation underwent liquid scintillation count, bars show mean counts of duplicate measurements with error bars showing the SD.

**Figure 2**

**0.25M sucrose**

**0M sucrose**


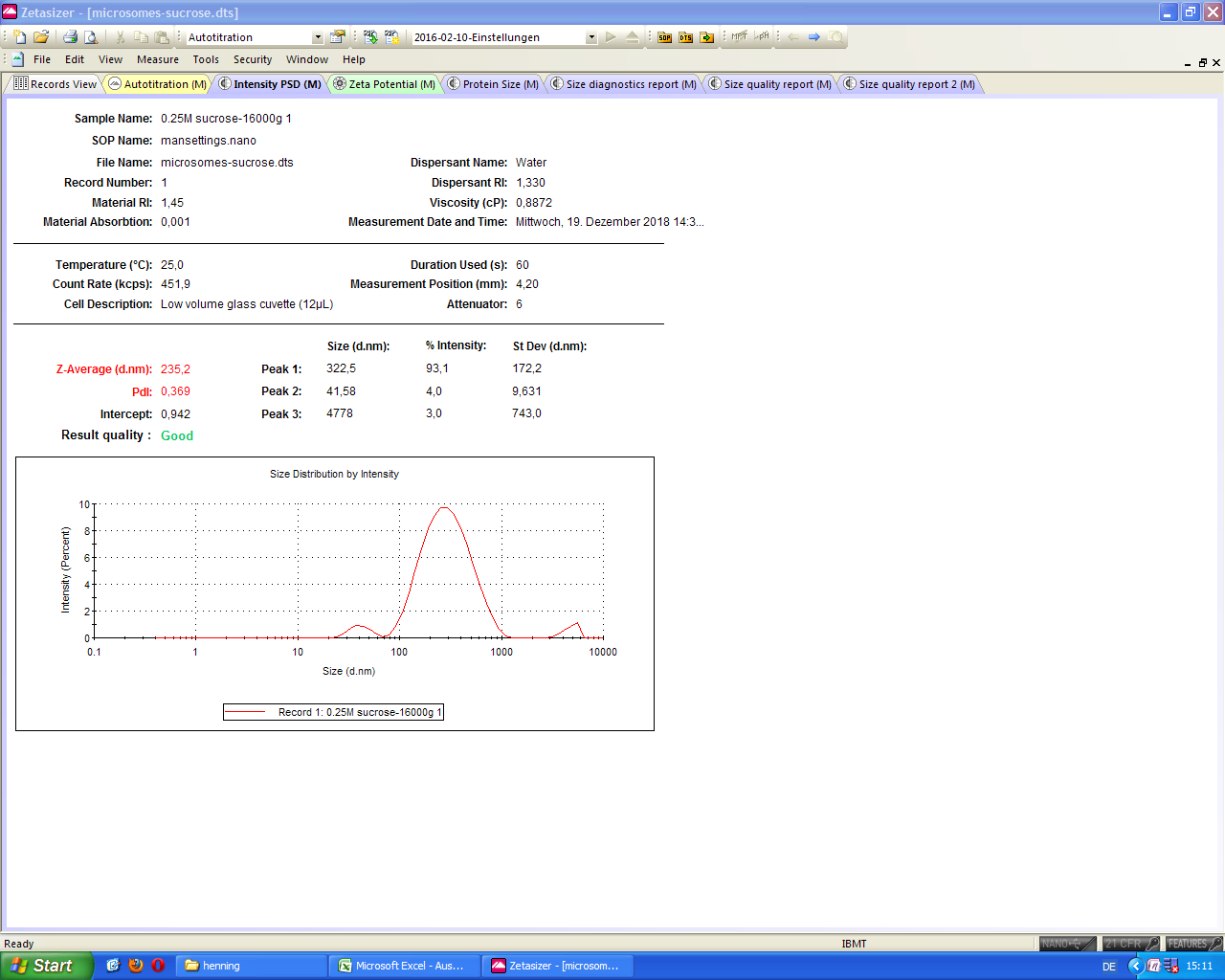

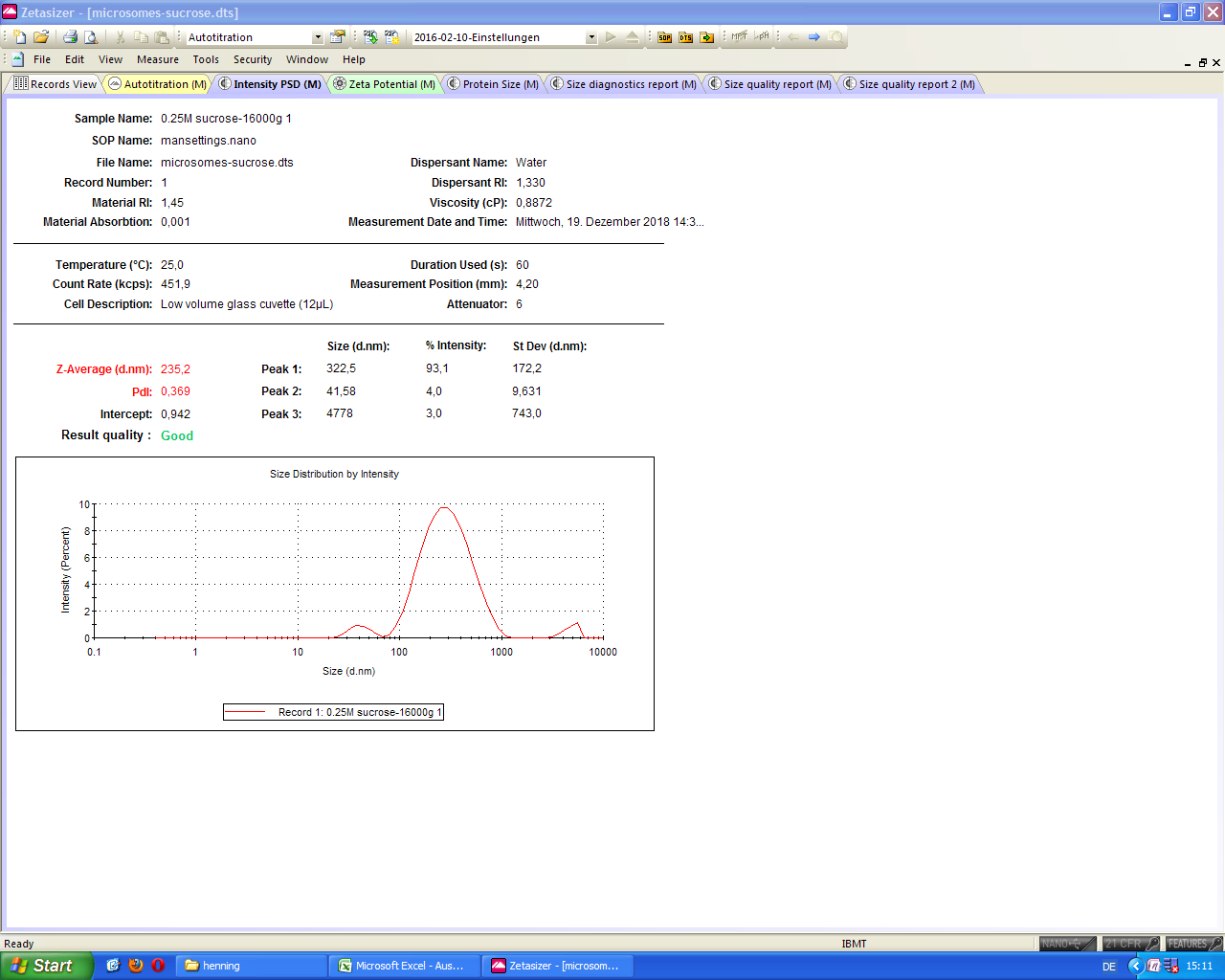

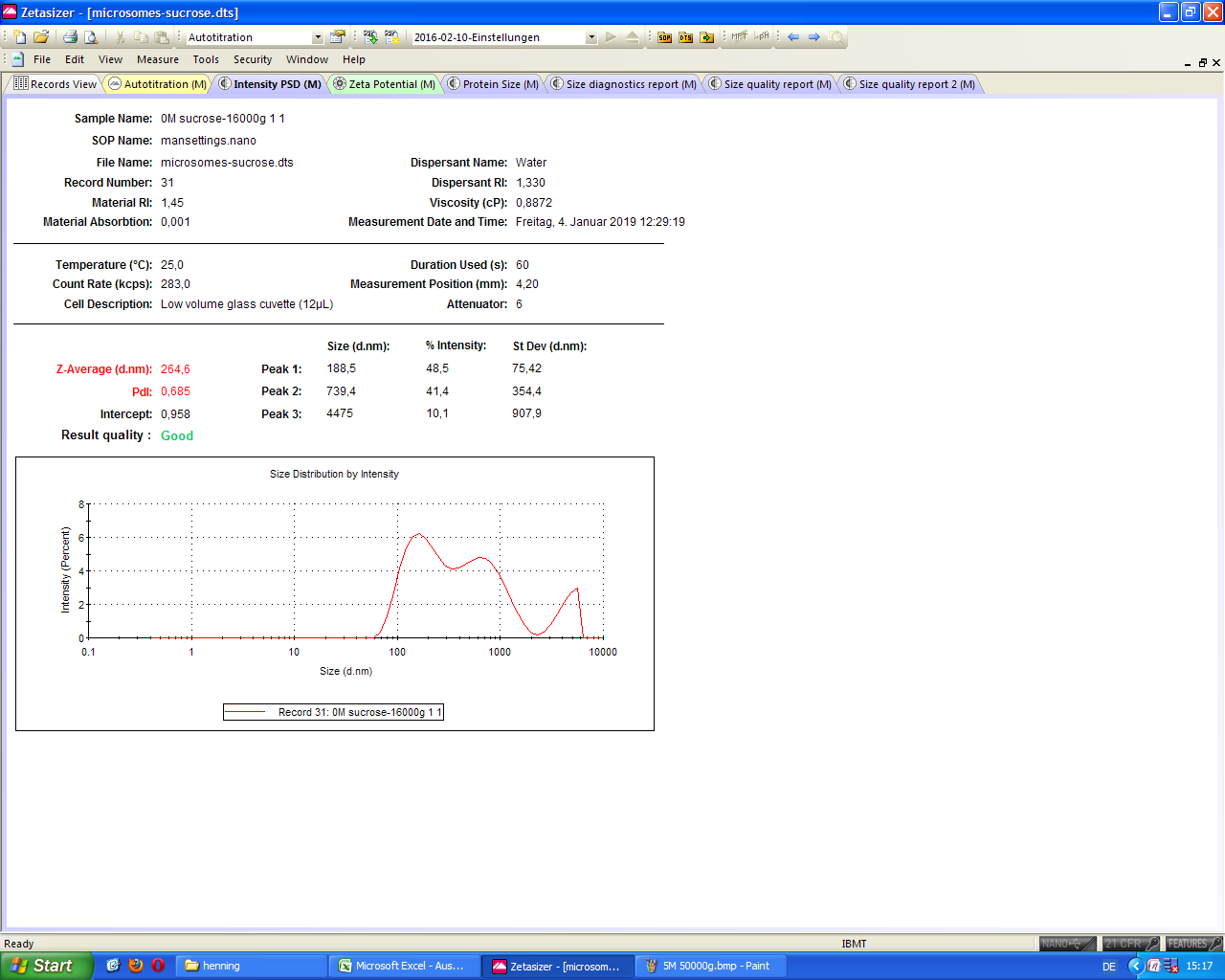

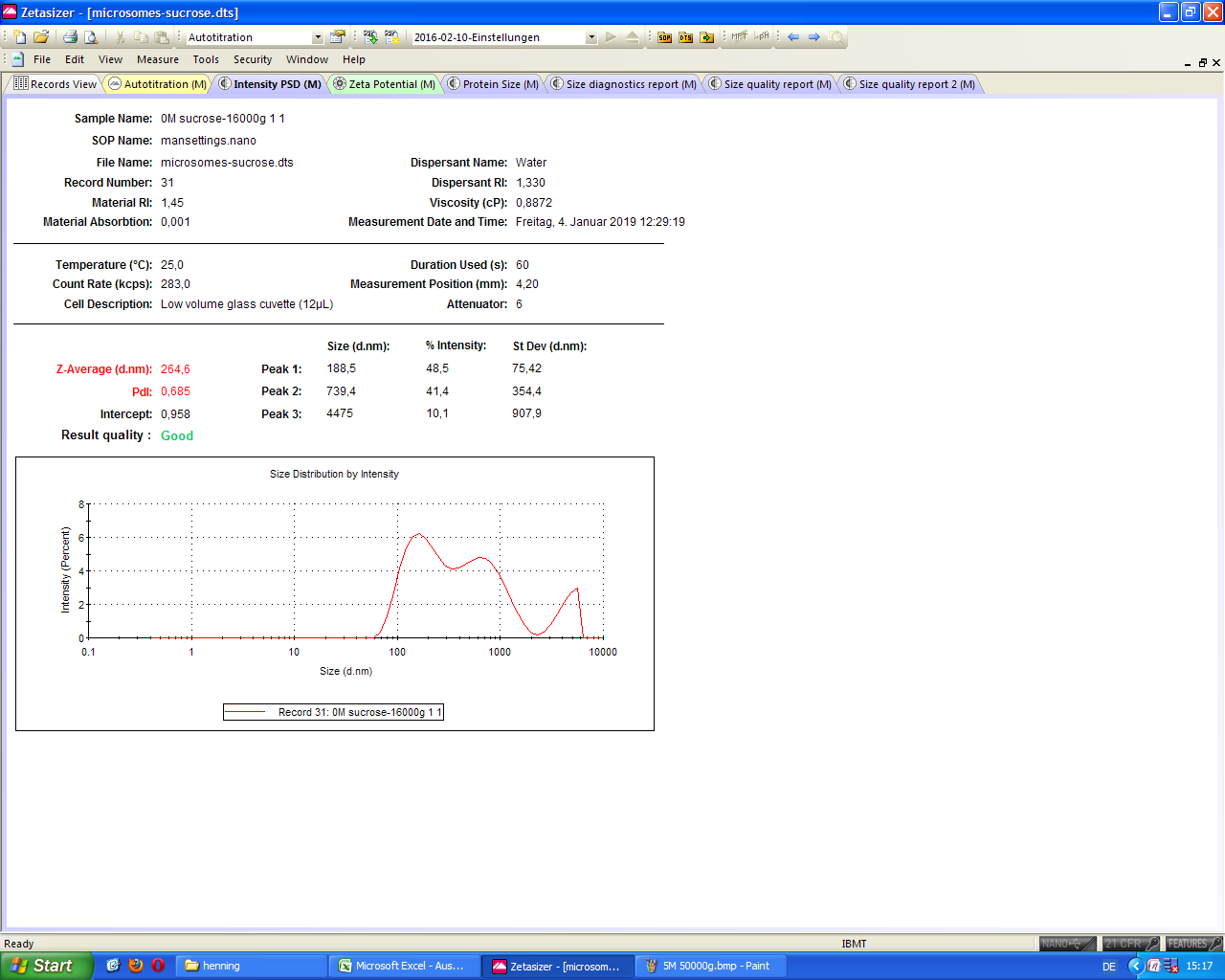


**2.5M sucrose**


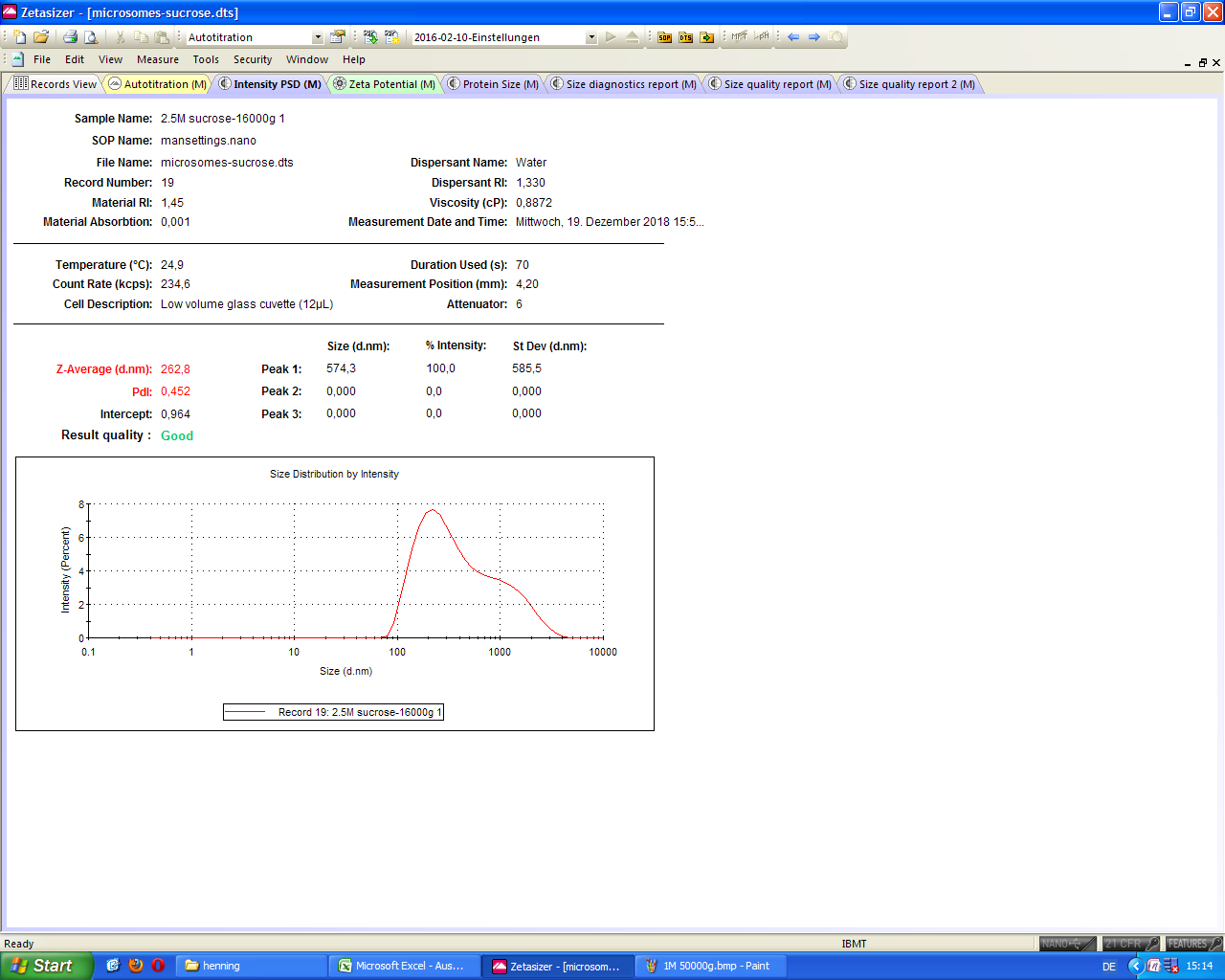

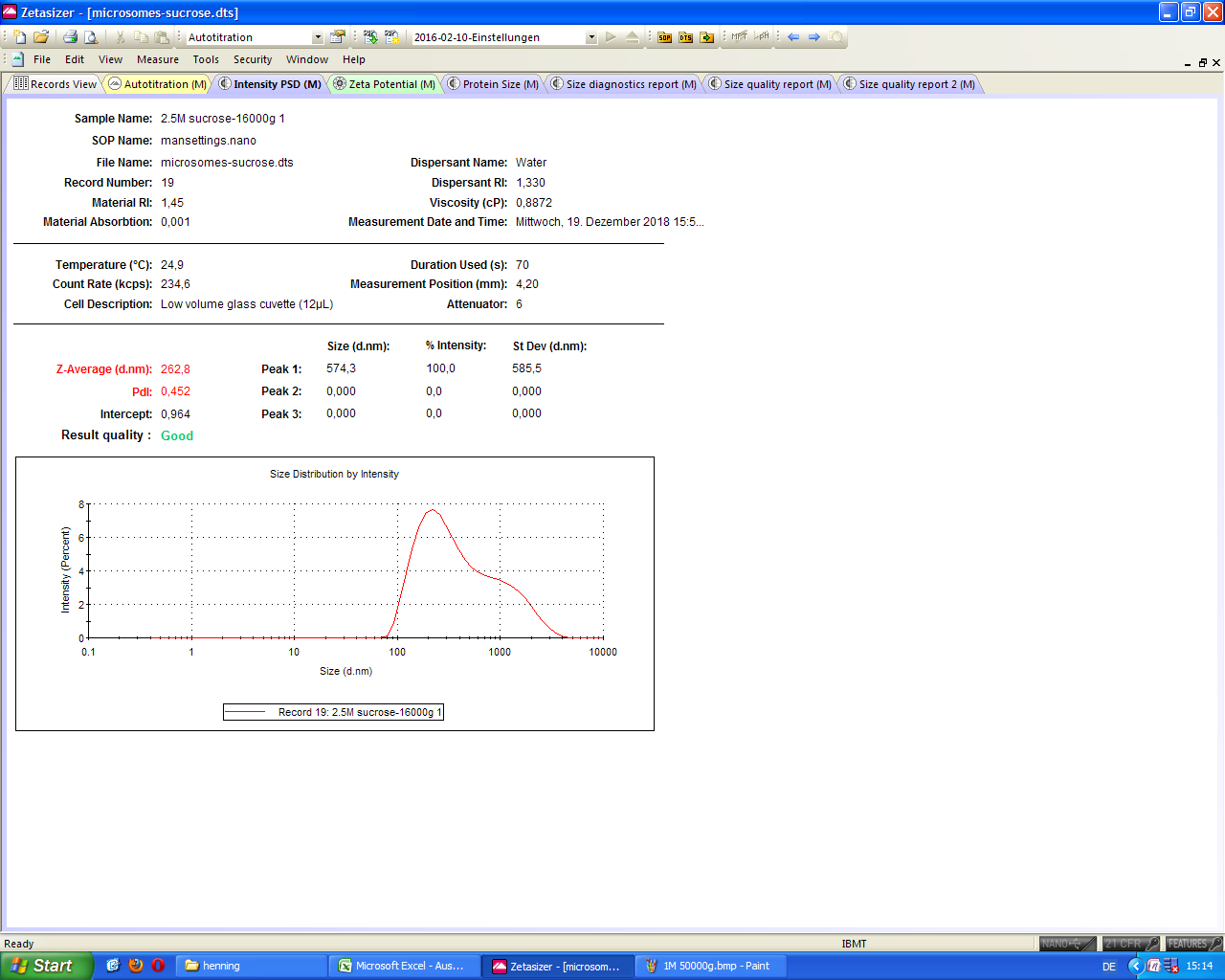


**Sup Figure 2. Vesicle diameter measurements of microsomal fractions after washing with different sucrose concentrations.** Microsomal fractions of *Sf*21 lysate were resuspended in uptake buffer with sucrose concentrations between 0 M and 2.5 M and subsequently measured with the zetasizer.
